# Genetic variability of three common NK and γδ T cell receptor genes (FCγ3R, NCR3, and DNAM-1) and their role in Polish patients with rheumatoid arthritis and ankylosing spondylitis

**DOI:** 10.1007/s12026-024-09488-3

**Published:** 2024-05-07

**Authors:** Sylwia Biały, Milena Iwaszko, Jerzy Świerkot, Katarzyna Kolossa, Joanna Wielińska, Sławomir Jeka, Katarzyna Bogunia-Kubik

**Affiliations:** 1 Laboratory of Clinical Immunogenetics and Pharmacogenetics, Hirszfeld Institute of Immunology and Experimental Therapy, Polish Academy of Sciences, Wroclaw, Poland; 2 Department of Rheumatology and Internal Medicine, Wroclaw Medical University, Wroclaw, Poland; 3 Clinical Department of Rheumatology and Connective Tissue Diseases, Jan Biziel Hospital University, No. 2, Bydgoszcz, Poland; 4 Ludwik Rydygier Collegium Medicum in Bydgoszcz, Nicolaus Copernicus University, Torun, Poland

**Keywords:** Rheumatoid arthritis, Ankylosing spondylitis, Polymorphism, NCR3, FCγR3A, DNAM-1, Anti-TNF treatment

## Abstract

Various lymphocyte subpopulations, including NK cells as well as γδ T cells, have been considered an important element in the pathogenesis of autoimmune, inflammatory, rheumatic diseases, such as rheumatoid arthritis (RA) and ankylosing spondylitis (AS). The aim of this study was to assess the potential role of polymorphic variations in the genes coding for three NK and γδ T cell receptors: NCR3, FCγR3A, and DNAM-1 (rs1052248, rs396991, and rs763361, respectively) in the disease susceptibility and the efficacy of treatment with TNF inhibitors. The study included 461 patients with RA, 168 patients with AS, and 235 voluntary blood donors as controls. The *NCR3* rs1052248 *AA* homozygosity prevailed in RA in patients lacking rheumatoid factor (*p* = 0.044) as well as in those who manifested the disease at a younger age (*p* = 0.005) and had higher CRP levels after 12 weeks of anti-TNF therapy (*p* = 0.021). The *FCγR3A* rs396991 polymorphism was associated with pain visual analogue scale (VAS) values before the initiation of anti-TNF treatment. Lower VAS values were observed in the *GG* homozygous RA patients (*p* = 0.024) and in AS patients with the *TT* genotype (*p* = 0.012). Moreover, AS heterozygous patients with the *TG* genotype presented higher CRP levels in the 12th week of anti-TNF treatment (*p* = 0.021). The findings suggest that the *NCR3* rs1052248 *AA* homozygosity may have an adverse effect on RA, while the *T* allele potentially plays a protective role in the development of AS. Moreover, the rs1052248 *T* allele and *TT* genotype appear to have a favorable impact on the response to anti-TNF therapy in RA patients.

## Introduction

Rheumatoid arthritis (RA) and ankylosing spondylitis (AS) belong to the commonly diagnosed autoimmune rheumatic diseases. It is estimated that rheumatic diseases affect about 0.9–1.5% of the world’s population [1]. Their perpetuation or implemented treatment can contribute to the development of comorbidities such as cardiovascular disease, cancer, lung disease, gastrointestinal ulcers, and various infections, and even lead to premature death [2–12].

The development of RA and AS is influenced by various risk factors, including environmental factors, lifestyle choices, history of infectious diseases, and socio-economic dimensions. Geo-epidemiological differences are also apparent, which may suggest the importance of factors related to patient ethnicity [13] and associated genetic variation.

One of the well-known and extensively studied genetic factors in RA is the so-called shared epitope (SE) which is a four-amino acid sequence (70–74 position) in the human leukocyte antigen (HLA)-DRB1 molecule. The presence of the shared epitope has a significant impact on the development and severity of RA [14] due to its potential role in CD4 + T lymphocyte activation. Simultaneously, HLA-B27 has been implicated in AS susceptibility. HLA-B27 belongs to HLA class I molecules and its main role is the presentation of antigens to CD8 + T lymphocytes leading to their activation. One of the widely accepted theories of HLA-B27’s role in the pathogenesis of AS is that HLA-B27 presents external bacterial antigens that resemble human antigens. As a result, CD8 + lymphocytes may be activated by self-antigens and propagate inflammation [15]. Both types of HLA molecules interact with αβ T cell receptors (TCR).

γδ T lymphocytes and natural killer (NK) cells have been implicated in the pathogenesis of rheumatic diseases, alongside T helper (CD4 +) and cytotoxic (CD8 +) cells [1, 16–19]. The role of γδ T cells in rheumatic diseases remains unclear and requires further exploration, although some reports suggest that they play a key role in disease pathogenesis. For instance, patients with RA have shown elevated levels of γδ T cells in synovial fluid compared to healthy individuals [20]. Furthermore, γδ T cells are known to produce interleukin (IL)-17, a cytokine involved in the pathogenesis of inflammatory diseases [16].

On the other hand, the role of NK cells in the development of rheumatic diseases has already been quite well described [18, 19]. It is known that the beneficial or detrimental effect of NK cell activity results from the balance of inhibitory and activating receptors on the surface of these cells.

Among a wide repertoire of surface receptors, there are three which have the ability to stimulate both γδ T cells as well as NK cells, namely NCR3, FCγ3R, and DNAM-1. These receptors are involved in the synergistic pathway of NK cell activation [21, 22].

The natural cytotoxicity triggering receptor 3 (NCR3) belongs to the immunoglobulin superfamily (IgSF) and is also referred to as a cluster of differentiation 337 (CD337) or natural killer protein 30 (NKp30) [23]. The gene for NCR3 is located on the human chromosome 6. Rusakiewicz et al. showed that NCR3 is a very important element in the mechanism of action of interferon-gamma (IFNγ), which has been identified as an important element in the pathogenesis of some autoimmune diseases, including RA [24–26]. In RA patients, increased levels of NCR3 were detected in the synovium, which was associated with more severe inflammation within the joints [26].

The A form of the FCγ3R receptor (encoded by *FCγR3A*) is one of the six-member human FCRs family, each with varying affinities for IgG immunoglobulins. FCγ3R is considered a low-affinity receptor [27] and is encoded by two genes *FC*γ*R3A* and *FC*γ*R3B* located on chromosome 1q23 sequentially along with other low-affinity FCR molecules. The FCR family has recently attracted considerable research interest due to their involvement in the development of various autoimmune and inflammatory diseases [28–30]. However, the exact role of FCRs in the development of rheumatic diseases has not been exhaustively described to date. FCRs are mostly produced by monocytes, macrophages, neutrophils, and NK cells involved in cytotoxicity, phagocytosis, and mediating pro-inflammatory molecule production [31, 32]. These receptors are also expressed on the surface of osteoclasts, which may suggest a role in the erosion of bone and cartilage structures within affected joints [33, 34]. One of the more recent studies discussing the mouse equivalent of FCγ3RA indicates its function in inducing the production of S100A8/S100A9 inflammation-mediating molecules that have been detected in the synovial fluid of arthritic joints [33]. They are mainly produced by neutrophils in whose activation FCγR3A is involved [35].

The DNAX accessory molecule-1 (DNAM-1), known also as Cluster of Differentiation 226 (CD226), is an activating receptor that is primarily involved in NK cell–dependent antitumor reaction and is also a very important element in the antiviral immune response [36–39]. It has been reported that the interaction between DNAM-1 and its ligand CD155 (also often described as poliovirus receptor, PVR, or Nectin-like molecule-5, Necl5) supports the polarization of T cells towards the Th1 subpopulation and the development of inflammation. On the other hand, the action of DNAM-1 results in inhibiting the JAK-STAT pathway by preventing phosphorylation, which reduces the Th2 lymphocyte response [40]. In addition, stimulated DNAM-1 can cooperate with the NCR3 receptor. Together, they enable communication between NK cells and dendritic cells and are involved in the proliferation of mature dendritic cells [41]. Moreover, previous studies have linked DNAM-1 with predispositions to rheumatic diseases, including RA [42–44].

The aim of our present study was to investigate the role of polymorphic variants within the genes encoding NCR3, FCγR3A, and DNAM-1 receptors in the development of inflammatory rheumatic diseases and the outcome of biological treatment with tumor necrosis factor (TNF) inhibitors. For this purpose, RA and AS patients and unrelated healthy controls were genotyped for two non-synonymous single nucleotide polymorphisms (SNPs): (I) a *T* to *G* substitution (rs396991) within the *FCγR3A* gene resulting in phenylalanine (Phe) to valine (Val) amino acid exchange in position 158 (Phe158Val), and (II) a *C* to *T* substitution (rs763361) within the *DNAM-1* gene resulting with glycine (Gly) to serine (Ser) amino acid exchange in position 307 (Gly307Ser), as well as (III) a *T t*o *A* substitution (rs1052248) in the 3′ UTR of the *NCR3* gene. To our knowledge, the role of these SNPs has not been previously investigated in Polish patients with RA and AS.

## Materials and methods

### Patients and controls — inclusion and exclusion criteria

A total of 461 RA patients and 168 AS patients were investigated. The basic condition for qualifying rheumatic patients for the study was high disease activity at the time of classification, non-response for treatment with the use of at least two conventional synthetic disease-modifying anti-rheumatic drugs (csDMARDs) for RA or non-steroidal anti-inflammatory drugs (NSAIDs) for AS patients. As a result, those patients were enrolled in the treatment program with TNF inhibitors. RA and AS activity was determined based on the American College of Rheumatology (ACR)/European League Against Rheumatism (EULAR) from 2010 and 1984 modified New York Criteria, respectively [45, 46]. Biological material was obtained in cooperation with the Department of Rheumatology and Internal Medicine, University Clinical Hospital in Wroclaw, Poland, and the Department of Rheumatology and Connective Tissue, Jan Biziel University Hospital No. 2 in Bydgoszcz, Poland.

All participants had to be over 18 years old, of Polish ethnicity, and lack other autoimmune and cancer diseases in their medical review. Moreover, they should be willing to cooperate. Failure to meet any of the above criteria resulted in the exclusion of the patient from the study group.

The control group consisted of 235 healthy voluntary blood donors. These individuals were over the age of 18 and belonged to the Polish ethnic group. The controls were not related to each other and had no medical history of immunological or hematologic diseases. All participants were of Caucasian ancestry. The study protocol was approved by the Wroclaw Medical University Ethics Committee.

### Selection of therapy and assessment of treatment effectiveness

Classified RA and AS patients were treated with one of the approved biological drugs that inhibit the effects of TNF. The dosage administered was determined by the doctor in accordance with the recommended guidelines for the respective medication. Additionally, methotrexate (MTX) was utilized as the primary first-line therapy for RA, and NSAIDs for AS were permitted for use.

Demographic data, including gender, age, and BMI, were collected from the patients. Clinical data such as C-reactive protein (CRP), rheumatoid factor (RF), and anti-cyclic citrullinated peptide (anti-CCP) antibodies (for RA patients) levels, as well as HLA-B27 status (for AS patients), were obtained through medical examinations prior to the initiation of TNF inhibitor therapy. Disease activity was assessed using the Disease Activity Score 28 (DAS28) for RA patients and the Bath Ankylosing Spondylitis Disease Activity Index (BASDAI) for AS patients. Additionally, patients self-reported their level of pain on a visual analogue scale (VAS). Subsequent medical examinations took place at 12 and 24 weeks after the initiation of TNF inhibitor therapy. During these medical appointments, additional clinical data were collected, and anti-TNF treatment effectiveness was assessed based on EULAR response criteria.

The condition of the patients was characterized by collecting and analyzing the parameters given in Table 1.

**Table 1:** Characteristics of RA and AS patients

Characteristics	RA	AS
Sex, female, *n* [%]	361 [78%]	45 [27%]
Age, mean [years] ± SD	51.71 ± 12.25	43.69 ± 13.51
Disease onset, mean ± SD [years]	39.16 ± 12.56	31.70 ± 9.96
BMI, mean ± SD	25.45 ± 5.17	25.56 ± 4.59
**Markers**		
RF positive [%]	68%	-
Anti-CCP positive [%]	91%	-
HLA-B27 positive [%]	-	90%
**Changes in clinical parameters during treatment**^1^		
CRP at baseline [mg/l] mean ± SD	23.40 ± 34.24	29.68 ± 55.05
ΔCRP [mg/l]; mean ± SD	15.61 ± 32.40	11.86 ± 25.48
DAS 28 at baseline mean ± SD	6.40 ± 0.68	-
ΔDAS 28 mean ± SD	3.31 ± 1.12	-
BASDAI at baseline mean ± SD	-	7.48 ± 1.38
ΔBASDAI mean ± SD	-	5.01 ± 2.57
**Treatment** [% of patients]		
Etanercept^3^	52.61%	29.17%
Adalimumab^4^	37.39%	40.48%
Inflliximab^5^	3.48%	1.19%
Certolizumab pegol^6^	6.52%	17.86%
Golimumab^7^	-	8.33%

*RA* rheumatoid arthritis, *AS* ankylosing spondylitis, *BMI* body mass index, *RF* rheumatoid factor, *Anti-CCP* anti-cyclic citrullinated peptide antibody, *HLA-B27* human leukocyte antigen B27, *CRP* C-reactive protein, *DAS 28* disease activity score 28, *BASDAI* Bath Ankylosing Spondylitis Disease Activity Index, *SD* standard deviation.

^1^The decrease in the level of parameters was calculated as the difference between pre-treatment data and the results after 24 weeks of treatment (0–24th week), and it was expressed as an absolute value.

^2^Doses selected individually for the patient with the maximum tolerated or recommended dose of 25–30 mg/week.

^3^Fifty milligrams administered by subcutaneous injection once a week.

^4^Forty milligrams administered by subcutaneous injection every other week.

^5^Three milligrams per kilogram (5 mg/kg for AS IV distributed at 0, 2, and 6 weeks as an intravenous infusions and then once every 8 weeks thereafter as a maintenance regimen.

^6^Four hundred milligrams (2 subcutaneous injections of 200 mg each) at weeks 0, 2, and 4, and then 200 mg every 2 weeks.

^7^Fifty milligrams administered by subcutaneous injection once a month.

### Polymorphisms selection

The* FCγR3A* rs396991 polymorphism is located on the 1st human chromosome in the low-affinity IIIA Fc fragment. It is a substitution from *T* to *G* which leads to changing phenylalanine (F) to valine (V) and is described also as *T* > *G*, Phe158Val, F176V, or F158V. This change probably leads to functional modifications related to the affinity of the FC*γ*R3A receptor for IgG [47]. Literature data have shown the relationship of this polymorphism with autoimmune diseases and their treatment [48–53].

The* NCR3* rs1052248 substitution is found on the 6th chromosome in the 3′ UTR gene sequence. The difference in variants is the change from *T t*o *A*. This polymorphism has been quite poorly examined in the context of rheumatic diseases, but the changing level of NCR3 protein during joint inflammation has been reported [26].

*DNAM-1* rs763361 is also described as Gly307Ser (*C/T*) in the CD226 gene located on the 18th chromosome in humans. Its relationship to rheumatic diseases has been pointed out in several papers, mainly in multiple sclerosis, rheumatoid arthritis, and systemic lupus as well [54–57]. Moreover, this polymorphism is likely involved in cellular signaling through the TCR pathway and activation of naïve T lymphocytes for proliferation and differentiation [58].

### Genotyping of receptor genes

Peripheral blood samples were collected on EDTA from RA and AS patients as well as from voluntary blood donors. DNA was isolated from the peripheral blood using the column isolation method (QIAamp DNA Blood Midi/Maxi Kit, Qiagen, Germany). The DNA samples were further subjected to analysis of three SNPs: *FCγR3A* rs396991 *(T* > *G*; Phe158Val); *DNAM-1* rs763361 (*C* > *T*; Gly307Ser); and *NCR3* rs1052248 (*A* > *T*). All selected polymorphisms were characterized with minor allele frequency (MAF) > 0.05, and are non-synonymous or are located within regulatory regions. Genotyping was performed with the use of commercial LightSNP kits employing real-time PCR (TIB MOLBIOL, Germany). Amplifications were carried out on the Real-Time PCR Instrument 480 (Roche Diagnostics, Switzerland).

### Statistical analysis

All non-normally distributed continuous data were subjected to analysis using the Mann–Whitney *U*-test. For data with a normal distribution, the parametric Student *T*-test with Welch’s correction for unpaired features was employed. Quantitative data were evaluated using Fisher’s test. Distribution was assessed using the Shapiro–Wilk test.

Statistical calculations and analyses were performed using GraphPad Prism 8.0.1 software, with results deemed statistically significant if the *p*-value was below 0.05.

## Results

### Genotype and allele distributions of investigated polymorphisms in RA and AS patients and control cohort

The distribution of alleles and genotypes for all selected polymorphisms was tested to check if they are in Hardy–Weinberg equilibrium (HWE). All groups satisfied the conditions of Hardy–Weinberg equilibrium.

Similarly, no significant difference was observed between both groups of the RA and AS patients and the controls concerning the *DNAM-1* rs763361 polymorphism.

However, an interesting difference was observed in the distribution of particular alleles/genotypes of the *NCR3* rs1052248 polymorphism between the group of patients suffering from AS and the control cohort. The *TT* genotype has not been identified among AS patients, whereas it occurred in healthy individuals with a frequency of approximately 10% (*p* < 0.0001; OR = 0.028, Table 2) suggesting a potential protective effect of this homozygous genotype against AS susceptibility. This relationship was not observed for RA patients.

**Table 2:** Distribution of studied polymorphism alleles and genotypes among control cohort and patients suffering from RA and AS. *p and *OR values were obtained from Fisher’s 2 × 2 test comparing the presence of a specific allele/genotype vs. other alleles/genotypes in the RA group compared to the healthy cohort. **p and **OR values were obtained from Fisher’s 2 × 2 test comparing the presence of a specific allele/genotype vs. other alleles/genotypes in the AS group compared to the healthy cohort

Polymorphism	Genotype	Controls *n* = 235 [%]	RA patients *n* = 461 [%]	*p-values	*OR (95% Cl)	AS patients *n* = 168 [%]	**p-values	**OR (95% Cl)
*FCγR3A* rs396991	*T*	310 [66%]	587 [64%]	0.442	0.900 (0.716; 1.142)	208 [69%]	0.330	1.167 (0.855; 1.592)
	*G*	160 [34%]	335 [36%]			92 [31%]		
	*TT*	108 [46%]	185 [40%]	0.145	1.269 (0.924; 1.742)	71 [47%]	0.834	0.946 (0.628; 1.427)
	*TG*	94 [40%]	217 [47%]	0.077	0.750 (0.545; 1.031)	66 [44%]	0.459	0.849 (0.560; 1.285)
	*GG*	33 [14%]	59 [13%]	0.723	1.113 (0.704; 1.760)	13 [9%]	0.147	1.722 (0.874; 3.390)
*NCR3* rs1052248	*A*	346 [74%]	687 [75%]	0.624	1.066 (0.827; 1.374)	289 [87%]	< 0.0001	2.302 (1.582; 3.349)
	*T*	124 [26%]	231 [25%]			45 [23%]		
	*AA*	133 [57%]	257 [56%]	0.940	1.025 (0.746; 1.407)	122 [73%]	0.001	0.481 (0.313; 0.738)
	*AT*	80 [34%]	173 [38%]	0.360	0.853 (0.614; 1.186)	45 [27%]	0.155	1.399 (0.905; 2.163)
	*TT*	22 [9%]	29 [6%]	0.194	1.532 (0.859; 2.730)	0 [0%]	< 0.0001	-
*DNAM-1* rs763361	*C*	256 [55%]	512 [56%]	0.740	1.039 (0.831; 1.300)	176 [54%]	0.885	0.972 (0.732; 1.291)
	*T*	212 [45%]	408 [44%]			150 [46%]		
	*CC*	70 [30%]	139 [30%]	1.000	0.986 (0.700; 1.390)	48 [29%]	1.000	1.023 (0.660; 1.585)
	*CT*	116 [50%]	234 [51%]	0.749	0.950 (0.693; 1.301)	80 [49%]	1.000	1.012 (0.684; 1.522)
	*TT*	48 [20%]	87 [19%]	0.685	1.106 (0.746; 1.641)	35 [22%]	0.900	0.944 (0.578; 1.541)

### Association of NCR3 rs1052248 polymorphism with clinical parameters in RA and AS patients

**Fig. 1:**
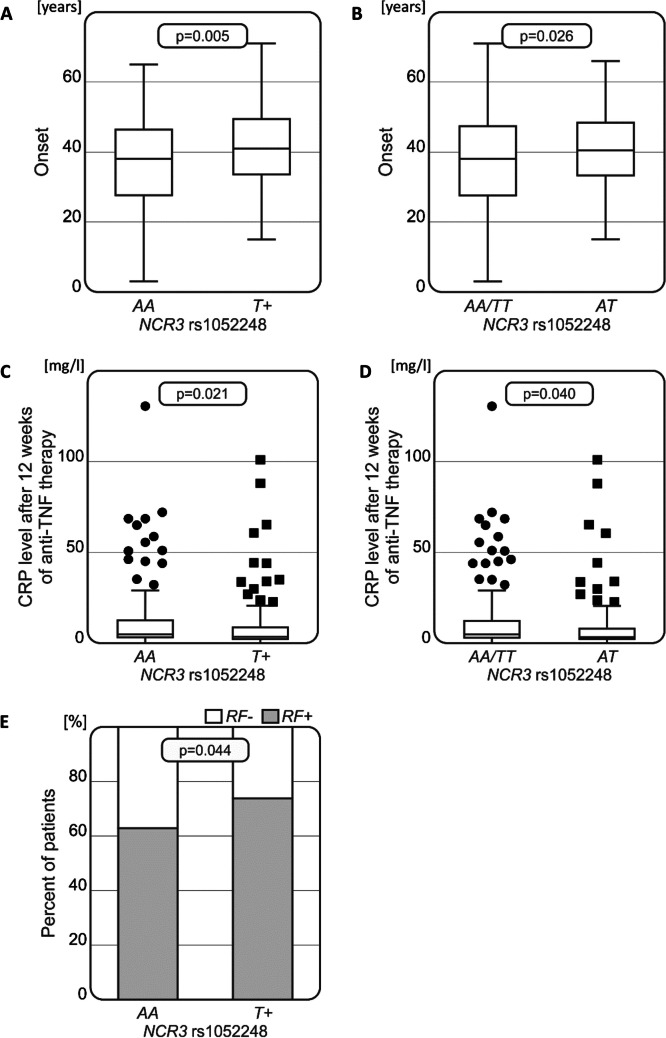
Associations between *NCR3* rs1052248 variants and clinical parameters in RA patients. Significant relationships between *NCR3* polymorphism and disease onset (**A**,** B**), CRP levels after 12 weeks of anti-TNF treatment (**C**, **D**), and the presence of RF in patients with RA (**E**)

Statistical analysis of results obtained from genotyping rs1052248 polymorphisms for the *NCR3* gene in relation to RA patients’ clinical parameters showed some significant relationships (Fig. 1).

The onset of RA was observed to be earlier in patients with the *NCR3* rs1052248 *AA* genotype than in carriers of the *T* allele (*p* = 0.005). Furthermore, the age of RA development was found to be affected by the presence of both *AA* and *TT* homozygous genotypes compared to *AT* heterozygosity, with *AA* and *TT* homozygous patients having onset at a younger age (*p* = 0.026) (Fig. 1A and B).

Moreover, *AA* homozygosity of the rs1052248 SNP was found to be associated with higher C-reactive protein (CRP) levels after 12 weeks of anti-TNF therapy (*p* = 0.021), while patients with the heterozygous *AT* genotype exhibited significantly lower blood CRP levels than homozygotes (*p* = 0.040) (Fig. 1C and D).

Additionally, the *AA* genotype was more frequently observed among RF-negative patients compared to patients with the *TT/AT* genotypes (*p* = 0.044, Fig. 1E).

No significant relationship was detected between the *NCR3* genetic variants and clinical parameters in patients with AS.

### Relationship between FCγR3A rs396991 polymorphism and VAS scale in RA and AS patients

**Fig. 2:**
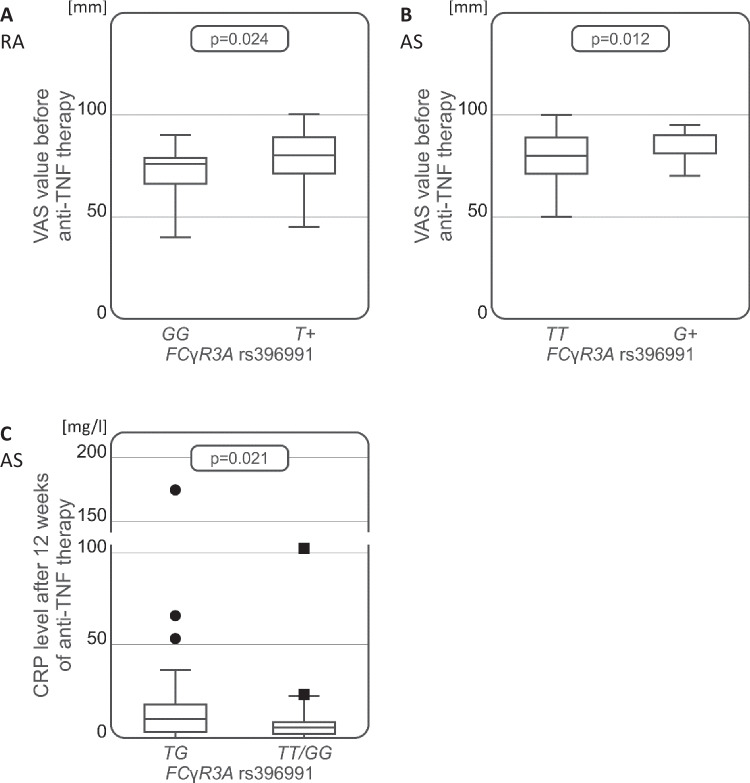
Associations between *FCγR3A* rs396991 variants and clinical parameters in RA and AS patients. Significant relationships between *FCγR3A* polymorphism and VAS values before initiation of anti-TNF treatment in RA (**A**) and AS patients (**B**), as well as CRP levels after 12 weeks of the anti-TNF treatment in patients with AS (**C**)

No association was observed between the *FCyR3A* rs396991 polymorphism and the risk of developing either RA or AS. However, some relationship was noted for this polymorphism concerning the VAS values before the initiation of anti-TNF therapy in both studied diseases. In the RA group, lower VAS values were observed in the *GG* homozygous patients than in those with other genotypes before starting anti-TNF therapy (*p* = 0.024, Fig. 2A). In the AS group, patients with the *G* allele marked higher pain values on the VAS scale than *TT* genotype carriers (*p* = 0.012, Fig. 2B). Moreover, among the group AS patients, the presence of heterozygous *TG* genotype was associated with higher CRP level than at the 12th week from the start of anti-TNF treatment as compared to patients carrying either *TT* or *GG FCγR3A* rs396991 homozygous genotype (*p* = 0.021, Fig. 2C).

Similarly to the *NCR3* rs1052248 and *FCγR3A* rs396991 polymorphism, the presence of the *DNAM-1* rs763361 genetic variant was tested for various clinical parameters in patients suffering from RA and AS. However, no significant relationships were observed.

## Discussion

The current state of knowledge highlights the significant role of NK cells and *γδ* lymphocytes in the pathogenesis and progression of rheumatic diseases [1, 18]. Intriguingly, these two cell types constitute an innate lymphoid population capable of producing inflammatory mediators and exerting cytotoxic activity. Furthermore, NK and γδ cells share surface receptors and transcription factors [59]. Therefore, we embarked on a study to investigate the impact of polymorphic variants in *NCR3*, *FCγR3A*, and *DNAM-1* genes, which are common to NK and γδ cells. Our objective was to explore their association with the development, progression, and response to TNF inhibitor treatment in patients diagnosed with rheumatoid arthritis and ankylosing spondylitis.

Our key findings concern the rs1052248 polymorphism of the *NRC3* receptor gene that was found to play a significant role in both groups of patients we examined. As mentioned earlier, it can be inferred from our study that the presence of the* T* allele seems to act as a protective factor of disease development, as observed for AS and RF-negative RA. This genetic variant was also less frequent among patients who developed RA at a younger age. Importantly, a more favorable impact of the rs1052248 *T* allele was also seen in RA patients concerning the anti-TNF treatment outcome, additionally confirming its beneficial effect. Notably, the rs1052248 polymorphism has been described for both the *NCR3* and *LTS1* genes, which are located close to each other and partially overlap, albeit they are transcribed in opposite directions on the complementary strands. These genes are situated within the MHC class III region, specifically in the Ltab-Ncr3 conserved haplotype, which has a significant association with arthritis pathogenesis and progression [60, 61]. Studies conducted on a rat model have demonstrated that increased *NCR3* expression and decreased *LST1* expression contribute to reducing the severity of RA. Similar results were obtained by the researchers when examining a group of 32 patients with RA [61].

The rs1052248 polymorphism within *LST1/NCR3* was also investigated in an intriguing study conducted by Wu et al. focusing on genome-wide interaction analysis in psoriasis, which is also an autoimmune disorder. While the primary aim of their work was to introduce a novel approach to the analysis of *locus* interactions, the findings presented demonstrate that the rs1052248 polymorphism in *LST1*/*NCR3* interacts with other polymorphisms, including the rs3131636 and rs3132468 single nucleotide substitutions in the *MICB* gene. Furthermore, the rs1052248 polymorphic site serves as a binding site for microRNA miR-324-3p [62]. It is noteworthy that different types of MIC family genes and miR-324 molecules have been linked in the literature to arthritis [63–66].

According to the literature data, the rs1052248 polymorphism of the *NCR3* gene has been very poorly investigated to date and there are no publications enabling us to compare the results of our present study with other cohorts of patients suffering from RA, AS, or other autoimmune diseases. In one of the published studies concerning the rs1052248 polymorphism, the authors examined its association with susceptibility to malaria in malaria-risk regions; however, no significant relationship was observed [67].

Nevertheless, numerous studies have shed light on the significant involvement of NCR3 in the progression of autoimmune conditions and the cytotoxic capabilities of cells [24, 68], undoubtedly contributing to the pathogenesis of RA and AS. Furthermore, the impact of the NCR3 protein has been observed in various other diseases, such as Sjögren’s syndrome and cancer-related disorders [24, 69].

In our present study, we also genotyped our RA and AS patients for the *FCγR3A* rs396991 polymorphism. The FCγR family of molecules has been associated with the pathogenesis of rheumatic diseases in many different studies [30, 70]. The primary role of FCγR molecules in the disease process is to recognize immune complexes and stimulate cells to produce inflammatory mediators in arthritic joints, which can further lead to the development of pain. Interestingly, our analyses showed some associations between the rs396991 polymorphism in the gene encoding the FCγR3A receptor and VAS scores in both groups of patients investigated, although their results are not consistent when comparing RA and AS patients. One study reported results quite similar to our own. Its authors pointed out that the presence of *FCγR3A* rs396991 *A* allele, as well as lower VAS values on the baseline, is in relation to better response to treatment by abatacept in RA patients. However, it is important to note that the VAS pain scale score is based on a subjective assessment determined by the patient [71]. Moreover, the results regarding the rs396991 *FCγR3A* polymorphism should be considered preliminary in nature as they are based on a small patient group. Nevertheless, these findings may provide insight into potential associations between genotype and clinical parameters in patients, particularly in the AS subgroup. It would be interesting to repeat this study on a larger patient cohort.

Furthermore, as in our study, the existing literature provides divergent perspectives on the identification of a beneficial variant of the rs396991 polymorphism in *FCγR3A* [50].

A meta-analysis conducted by Lee et al. demonstrated an association between the presence of the *GG* genotype and the occurrence of RA among Europeans but not Asians [72]. Several studies have examined the relationship between the polymorphism in the *FCγR3A* gene and the response to treatment with TNF inhibitors. It has been suggested that individuals with the homozygous dominant *TT* genotype have slower drug metabolism, leading to therapeutic benefits [73]. Similar findings were reported by Tutuncu et al. in a mixed American population [74] and Márquez Pete et al. in a Spanish population [71]. On the other hand, no association between treatment response and the specific polymorphic variant was found in either Swedish individuals [75] or in the Dutch population [76]. However, in our AS patients, the *FCγR3A* rs396991 heterozygosity seems to have an unfavorable impact on anti-TNF treatment with no effect of the *FCγR3A* rs396991 SNP in the RA patient group. In Spanish patients with AS, the *G* allele was reported to be associated with a better response to anti-TNF treatment. In the same study, Morales-Lara et al. also examined patients with RA and PsA, but the results did not align with those for AS, leading the authors to conclude that the drug’s impact may vary among different types of arthritic diseases, which we agree with [77].

The results of the third SNP studied, rs763361 polymorphism in the *DNAM-1* gene, did not show any significant association in either RA or AS patients of Polish origin. Nevertheless, in many other populations, this polymorphism has also not been unequivocally identified as a diagnostic or clinically significant factor, both in the diseases we investigated (RA and AS) and other autoimmune disorders [78–80]. One study involving an Iranian population suggests a possible negative association of the* T* allele, which increases the risk of developing RA [81], and meta-analyses encompassing diverse populations indicate some probability of autoimmune disease prediction, including for RA, using the rs763361 polymorphism in the *DNAM-1* gene [82, 83]. Furthermore, the study by Tan et al. indicates an association between the rs763361 polymorphism of *DNAM-1* and the response to TNF inhibitor treatment in RA [56]. However, our findings do not confirm this association.

## Conclusion

In summary, our results represent the first research study investigating the significance of the rs1052248 *NCR3,* rs396991 *FCγR3A*, and rs763361 *DNAM-1* polymorphisms in the pathogenesis, progression, and response to therapy with TNF inhibitors in Polish patients with rheumatoid arthritis and ankylosing spondylitis. To the best of our knowledge, the most promising results of our current study regarding the *NCR3* gene polymorphism provide new insights not only in the context of the Polish population but also as the only published results of their kind. We believe that all the polymorphisms we investigated are worth further attention and analysis, with a special focus on the rs1052248 *NCR3* polymorphism, which has the potential to become a prognostic factor for the more severe RA, as well as a predictor for AS, as observed in our Polish population.

## Data Availability

The data supporting the findings of this study are available within the article and available from the corresponding author on request.
